# Total Antioxidant Capacity of Some Commercial Fruit Juices: Electrochemical and Spectrophotometrical Approaches

**DOI:** 10.3390/molecules14010480

**Published:** 2009-01-20

**Authors:** Aurelia Magdalena Pisoschi, Mihaela Carmen Cheregi, Andrei Florin Danet

**Affiliations:** 1 Chemistry and Biochemistry Department, Faculty of Veterinary Medicine, University of Agronomic Sciences and Veterinary Medicine, bulevardul Marasti, no 59, Bucharest, Romania; E-mail: apisoschi@yahoo.com (A-M. P.); 2 Analytical Chemistry Department, Faculty of Chemistry, University of Bucharest, Panduri Avenue 90-92, code 050657, Bucharest, Romania

**Keywords:** Antioxidant activity, 2,2-Diphenyl-1-picrylhydrazyl, 2,2-Diphenyl-1-picrylhydrazine, Biamperometry, Pt electrode, Citrus juices.

## Abstract

The aim of this paper was to assess the total antioxidant capacity of some commercial fruit juices (namely citrus), spectrophotometrically and by the biamperometric method, using the redox couple DPPH· (2,2-diphenyl-1-picrylhydrazyl)/DPPH (2,2-diphenyl-1-picrylhydrazine). Trolox^®^ was chosen as a standard antioxidant. In the case of the spectrophometric method, the absorbance decrease of the DPPH· solution was followed. For the biamperometric method, the influence of some parameters like the potential diference, ΔE, DPPH· concentration, and Trolox^®^ concentration was investigated. The calibration graph obtained for Trolox^®^ presents linearity between 5 and 30 µM, (y = 0.059 x + 0.0564, where y represents the value of current intensity, expressed as μA and x the value of Trolox^®^ concentration, expressed as μM; r^2^ = 0.9944). The R.S.D. value for the biamperometric method was 1.29% (n = 10, c = 15 μM Trolox^®^). In the case of the spectrophotometric method, the calibration graph obtained for Trolox^®^ presents linearity between 0.01 and 0.125 mM (y = -9.5789 x+1.4533, where y represents the value of absorbance and x, the value of Trolox^®^ concentration, expressed as mM; r^2^ = 0.9963). The R.S.D. value for the spectrophotometric method was 2.05%. Both methods were applied to total antioxidant activity determination in real samples (natural juices and soft drinks) and the results were in good agreement.

## 1. Introduction

Oxidation is one of the most important free radical-producing processes in food, chemicals and even in living systems. Free radicals play an important role in food and chemical material degradation, contributing also to more than one hundred disorders in humans [1,2,3,4,5,6]. Highly reactive free radicals and oxygen species present in biological systems can oxidize nucleic acids, proteins and lipids, initiating degenerative diseases [7,8]. Antioxidants significantly delay or prevent the oxidation of easily oxidable substrates. 

Plants contain high concentrations of numerous redox-active antioxidants, such as polyphenols, carotenoids, tocopherols, glutathione, ascorbic acid and enzymes with antioxidant activity, which fight against hazardous oxidative damage of plant cell components. In animal cells, antioxidant production is much more limited and oxidative damage is involved in the pathogenesis of most chronic degenerative diseases (including cancer and heart diseases) and aging [9,10,11]. Therefore, plant-sourced food antioxidants like vitamin C, vitamin E, carotenes, phenolic acids, phytates and phytoestrogenes have been recognized as having the potential to reduce disease risk. 

The intake of food rich in *α*-tocopherols, *β*-carotene and ascorbic acid has been associated with reduced oxidative-stress related diseases. Phenolic acids, polyphenols and flavonoids scavenge free radicals such as peroxide, hydroperoxide or lipid peroxyl, thus inhibiting the oxidative mechanism that lead to degenerative diseases [12,13,14,15]. 

Many analytical methods have been used for antioxidant monitoring, for instance phenolics in fruit have been monitored by HPLC [16,17] or colorimetrically using the Folin Ciocalteu reagent [18]. The total antioxidant capacity of foods and plant extracts has been assessed by using spectrophotometric methods with DPPH· (2,2-diphenyl-1-picrylhydrazyl) [19,20,21], ABTS^+^· (2,2’-azinobis(3-ethylbenzo-thiazoline-6-sulfonic acid)) [19,20] or Fe^3+^-TPTZ (2,4,6-tripyridyl-*s*-triazine) [22]. 

The antioxidant content and total antioxidant activity were also evaluated electrochemically, by means of voltammetric or amperometric methods. Phenolics were determined by HPLC and FIA with amperometric detection [23], amperometric biosensors [24] and voltammetric techniques [25]. Biamperometric determination of the total antioxidant capacity uses redox couples like DPPH·/DPPH [26] or ABTS·^+^/ABTS, where the ABTS radical cation is bienzymatically produced by glucose oxidase and peroxidase, immobilized in a flow-through reactor [27]. Cyclic voltammetry results of antioxidant capacity determination in buckwheat products showed good correlation with the data obtained by spectrophotometry with DPPH· [28]. 

The aim of this work is to perform a comparative study regarding the determination of total antioxidant capacity of some fruit juices, by using the spectrophotometric method with DPPH· and biamperometry with a DPPH·/DPPH redox couple.

**Figure 1:**
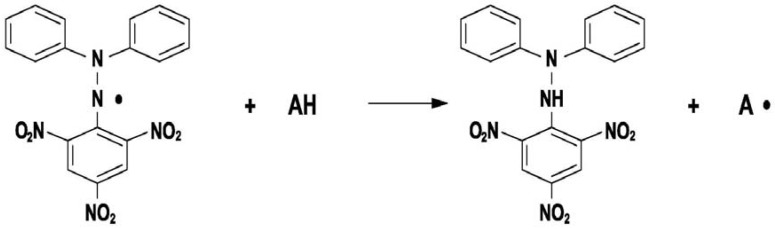
Reaction of DPPH· free radical with an antioxidant.

*Spectrophotometry–principle of the method:* the spectrophotometric method for assessing the total antioxidant activity is based on the absorbance decrease monitoring of the DPPH· radical (2,2-diphenyl-1-picrylhydrazyl) in the presence of antioxidants. DPPH· is characterized as a stable free radical due to the delocalization of the spare electron over the molecule. Thus, the molecule cannot dimerise, as would happen with other free radicals. The delocalization gives rise to a deep violet colour characterized by an absorption band at about 520 nm. When a DPPH· solution is mixed with a substance which can donate a hydrogen atom (see Figure 1), the reduced form is generated, accompanied by the loss of the violet colour [19,21,29]. 

*Biamperometry–principle of the method:* the biamperometric method is based on the measurement of the current flowing *between* two identical Pt working electrodes polarized at a small potential difference and immersed in a solution containing a reversible redox couple. Indirect biamperometric measurement relies on reaction of the analyte with the indicating redox couple, its selectivity depending on the specificity of the reaction involving the oxidized or reduced form of the redox pair and the analyte. Fe^3+^/Fe^2+^, I_2_/I^-^, Fe(CN)_6_^3-^/Fe(CN)_6_^4-^ are redox couples commonly used in biamperometric measurements. The redox pair chosen in this study was DPPH·/DPPH. Antioxidants react with DPPH· (radical form) generating DPPH (reduced form), like in the reaction presented in Figure 1, the intensity of the resulted current being proportional to the residual concentration of DPPH· after its reaction with the analyte (antioxidant). 

In the present study, we used two identical Pt electrodes where the reduction of the DPPH· radical and oxidation of the reduced form (DPPH) take place as follows:

*Electrode 1:* DPPH· + e^-^ → DPPH *Electrode 2:* DPPH → DPPH· + e^-^

The reduction of DPPH· at electrode 1 gives rise to a cathodic current, while the oxidation of DPPH at electrode 2 generates an anodic current. The biamperometric detector response is linear for that constituent of the redox couple which is present in lower concentration. Working conditions were chosen for a DPPH· concentration smaller than DPPH concentration. Each antioxidant addition in a solution containing the couple DPPH·/DPPH decreases the concentration of the oxidized (radical) form and increases the concentration of the reduced form, thus generating a current proportional to the concentration of antioxidant. In the case of the proposed method, cathodic current is limited by the lower concentration of DPPH· radical in the indicating mixture [26].

## 2. Results and Discussion

### 2.1. Spectrophotometry: the study of DPPH· absorbance decrease in the presence of antioxidants

Following the explained working procedure (see Experimental), the value of the absorbance diminishes as the antioxidant concentration increases because more DPPH· is quenched by Trolox^®^. It can be noticed that the absorbance signal for blank reached the steady state two minutes after Trolox^®^ addition and therefore the following readings were done at this time point.

**Figure 2:**
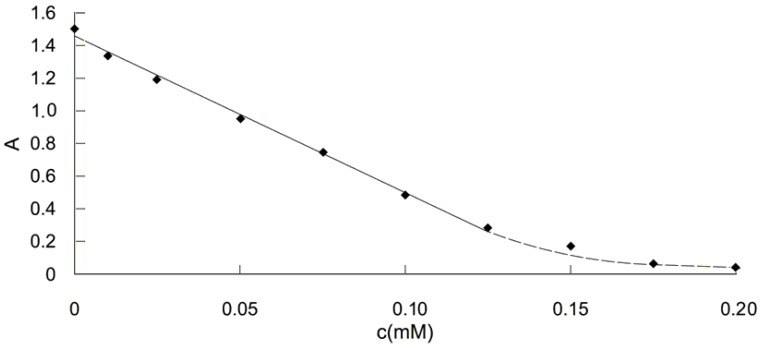
Absorbance variation for DPPH· 0.12 mM, in the presence of different Trolox^®^ concentrations.

The calibration graph (Figure 2) is linear in the range 0.01 to 0.125 mM for Trolox^®^, with an equation of y = -9.5789 x + 1.4533 (where y represents the value of absorbance and x, the value of Trolox^®^ concentration, expressed as mM) and a correlation coefficient of r^2^ = 0.9963. The flattening of the graph which begins at 0.150 mM Trolox^®^ concentration is a consequence of DPPH· almost complete quenching by Trolox^®^. The value of inhibition (Q%, defined as: 100x(A_0_-A_c_)/A_0_, where A_0_ represents the absorbance for blank and A_c_, the absorbance at different values of Trolox^®^ concentration) calculated for 0.150, 0.175 and 0.200 mM Trolox^®^ on the final solution were 89.00, 95.86% and 96.66%, respectively.

### 2.2. Electrochemical method

#### 2.2.1. Study of the reversibility of the DPPH·/DPPH redox couple

**Figure 3:**
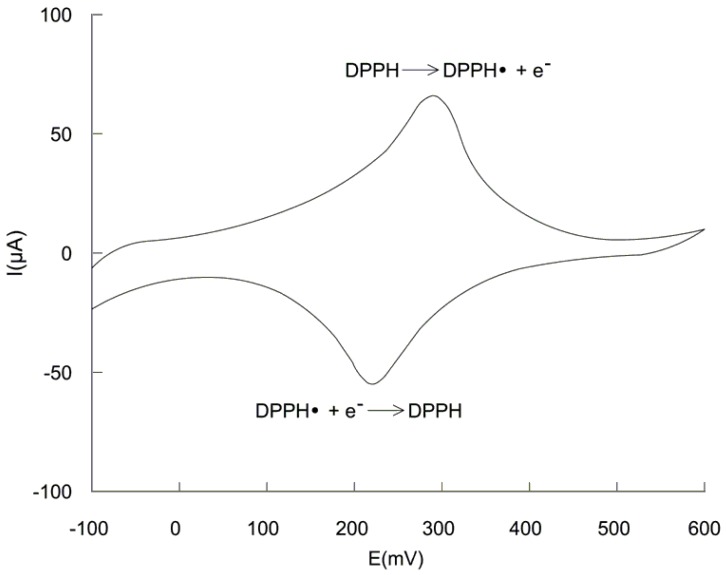
Cyclic voltammogram obtained for the DPPH·/DPPH redox couple; conditions: DPPH· concentration = 0.5 mM; DPPH concentration = 0.5 mM; potential sweep speed = 50 mV/s; (for details see Experimental).

To verify the reversibility of the redox DPPH·/DPPH couple, cyclic voltammetry experiments were performed with a three electrode electrochemical cell equipped with two Pt strip electrodes (30 mm^2^ surface) and a saturated calomel electrode as reference. The potential was swept from -100 to 600 mV. The registered voltammogram (Figure 3) shows the characteristics of a reversible redox couple, that is a 59 mV difference between the anodic peak corresponding to DPPH oxidation and cathodic peak corresponding to DPPH· reduction. 

#### 2.2.2. Study of the influence of the potential difference, ΔE, applied between the two electrodes on the analytical signal

**Figure 4:**
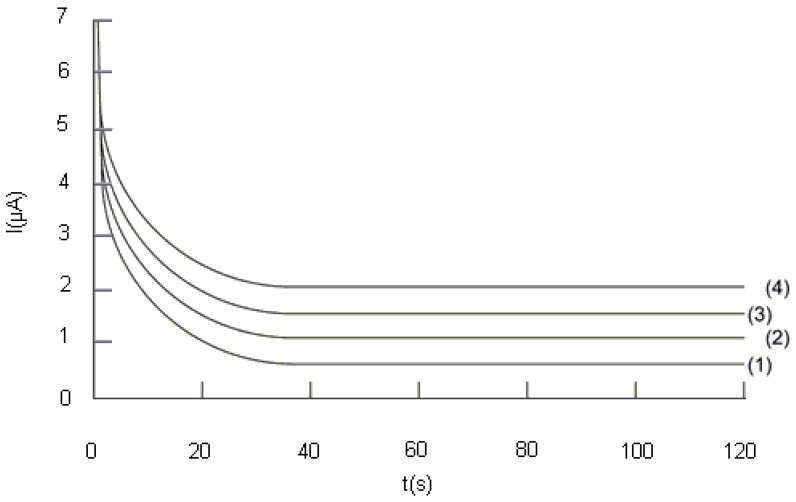
Chronobiamperograms illustrating the influence of the potential difference, ΔE: (1) 50 mV, (2) 100 mV, (3) 150 mV, (4) 200 mV; conditions: DPPH· concentration = 100 µM, DPPH concentration = 110 µM; (for details see Experimental).

**Figure 5:**
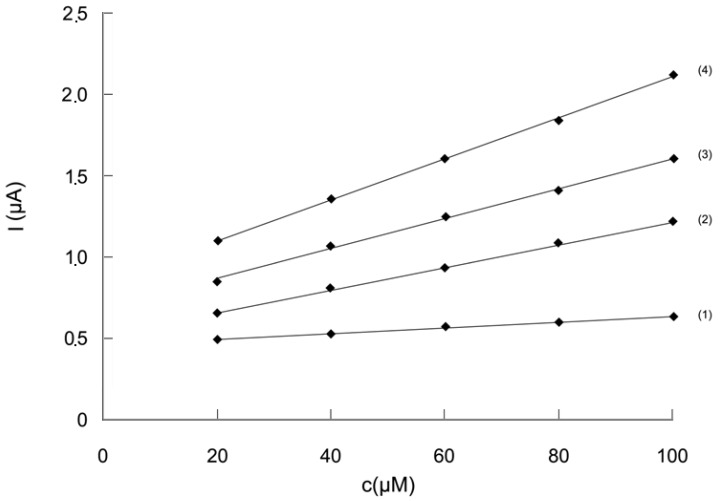
The analytical response plotted against DPPH· concentration at different values of ΔE: (1) 50 mV, (2) 100 mV, (3) 150 mM, (4) 200 mV; DPPH concentration = 110 µM. (for details see Experimental).

The influence of the potential difference, ΔE, applied between the two electrodes, on the analytical signal was studied and a series of chronoamperograms were registered in the two-electrode electrochemical cell (see Figure 4). The value of the current intensity obtained at different values of ΔE and read after one minute, was plotted against DPPH· concentration (Figure 5). By analysing the data plotted in Figure 4 and Figure 5, we notice that the current value on the plateau due to DPPH oxidation and DPPH· reduction, increases with the potential difference, ΔE.

The results presented in Figure 5 prove that the analytical signal is the greatest at 200 mV potential difference. Moreover, a proportionality can be noticed between current intensity and DPPH· concentration. Therefore, this value of the potential difference was chosen for investigating the influence of the Trolox^®^ concentration and real sample analysis. Greater values of the potential difference were not taken into account, because of electrode reactions which can occur at the electrode surface, involving other compounds present in the analysed sample, thus giving rise to interferences. 

#### 2.2.3. Study of the influence of the Trolox^®^ concentration on the analytical signal

**Figure 6:**
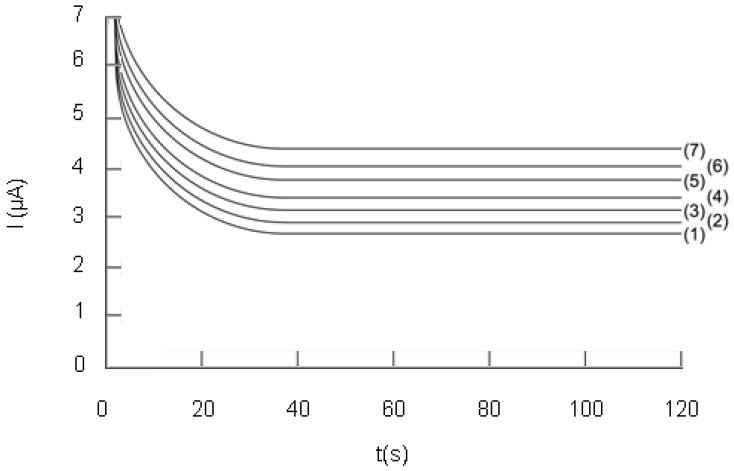
Chronobiamperograms obtained for different Trolox^®^ concentrations: (1) 0 µM, (2) 5 µM, (3) 10 µM, (4)-15 µM, (5) 20 µM, (6) 25 µM, (7) 30 µM; conditions:110 µM DPPH/100 µM DPPH·, potential difference, ΔE = 200 mV (for details see Experimental).

Increasing concentrations of Trolox^®^, from 5 to 30 µM, were added to the DPPH·/DPPH mixture solution and the obtained chronoamperograms are presented in Figure 6. 

**Figure 7:**
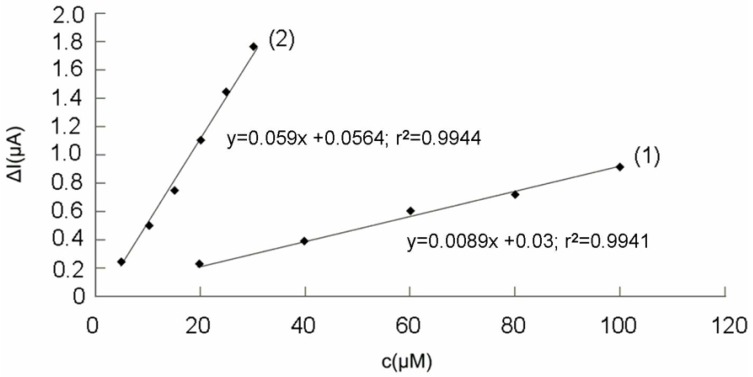
Analytical response of the biamperometric detector plotted against DPPH· concentrations (1) for 110 μM DPPH and ΔE = 200 mV and Trolox^®^ concentrations; (2) for 100 μM DPPH· and 110 μM DPPH, ΔE = 200 mV (for details see Experimental).

The analytical signals obtained for different concentrations of DPPH· and a concentration of 110 μM DPPH, as well as for different Trolox^®^ concentrations, in the presence of 100 μM DPPH· and 110 μM DPPH were represented in Figure 7. The biamperometric detector responses were read at 60 seconds. In both cases, the value of the intensity registered for 0 μM DPPH· or 0 μM Trolox^®^ concentration (blank) was substracted from the analytical signal obtained at different concentrations of DPPH or Trolox^®^, respectively. The obtained ΔI values were plotted against DPPH· and Trolox^®^ concentrations (see Figure 7). 

The slope difference between the two graphs presented in Figure 7 can be explained in terms of the stoichiometry of the reaction DPPH·/Trolox^®^ and the way the solvent affects DPPH· concentration, as reported previously [26,29,30]. Molyneux [21] also observed a difference between DPPH· theoretical (stoichiometrical) concentration and the one reacting with ascorbic acid. 

According to the experimental data plotted in Figure 7, 100 µM DPPH· is equivalent to approximately 20 µM Trolox^®^, which corresponds to a approximate 5/1 ratio. Considering 2/1 stoichiometry for the reaction between DPPH· and Trolox^®^ [29], that implies a difference between calculated DPPH· and the one determined in the experiment. The result is a 60 µM difference between prepared DPPH· concentration (100 µM) and the one reacting with Trolox^®^ (40 µM). This discrepancy is confirmed by the results found by other authors for the reaction DPPH·/Trolox^®^ and it can be explained by the fact that the solvent (ethanol) affected active DPPH· concentration [26,30]. Milardovic *et al.* [30] found a 40 µM difference between prepared DPPH··and the one determined in the biamperometric experiment. The spectroscopic calibration curve for Trolox^®^, obtained using 200 µM DPPH· in ethanol-water solution shows the same equivalent concentration range as established by the calibration curve for Trolox^®^, determined amperometrically [30]. The same equivalent concentration of DPPH in the radical form, determined spectrometrically and electrochemically implies that the active DPPH· concentration was affected by the solvent, which agrees with other literature data [21]. In addition to that, in the case of ethanol, solvent properties, such as the ability to form hydrogen bonds with the antioxidant, influence the level of the relative activity towards DPPH·[31]. 

The accuracy of biamperometric measurements of Trolox^®^ was excellent, RSD% = 1.29% (c = 15 µM Trolox^®^, n = 10). The detection limit (calculated as 3 x the standard deviation of the blank signal) was 0.0768 µM, whereas the limit of quantification (calculated as 10 x the standard deviation of the blank signal) was 0.256 µM. The Trolox^®^ calibration curve [Figure 7 (2)] was employed for the analysis of real samples (commercial, as well as juices obtained by fruit squeezing). 

### 2.3. Analysis of real samples

Natural juices obtained by squeezing fruit, which contained fruit pulp, needed previous centrifugation and 10-fold dilution with distilled water, while clear soft drinks did not necessitate any special sample preparation before analysis. 

Spectrophotometry: For soft drinks, the dilution degree varied between 1/2 and 1/50, while for juices obtained by pressing fruit greater dilution degrees are required, between 1/100 and 1/150. The working procedure used for standard antioxidant solutions (Trolox^®^) was also applied to fruit juices. 

The biamperometric method was also applied for total antioxidant capacity determination in juice real samples. For soft drinks, the dilution degree was comprised between 1/5 and 1/400, whereas for juices obtained by pressing fruit, the dilution degree ranged between 1/500 and 1/750. The experimental conditions and working procedure were the same as those used for the standard antioxidant, Trolox^®^. The obtained results are presented in Table 1. The highest values of TEAC (Trolox Equivalent Antioxidant Capacity) were registered for natural juices obtained by squeezing fruit. 

The previously mentioned results also prove that the greatest TEAC values are obtained for natural products: juices obtained by pressing fruit and fruit extracts. 

**Table 1:** Results, expressed as TEAC obtained for total antioxidant activity determination in fruit juices by spectrophotometry and biamperometry and comparison with literature data.

	Spectrophotometry		Biamperometry		Literature Data
*Product*	*TEAC value (spectrophotometry) mM Trolox*	*Absorbance value*	*TEAC value (biamperometry) mM Trolox*	*ΔI value (biamperometry)* *µA*	*TEAC values (mmoles Trolox/kg or mM Trolox)*
Orange juice	9.25	0.89	9.07	0.64	8.74 mmoles/kg fresh weight orange by ABTS+. method [13]
					8.88 mM sweet oranges extract^*^ by DMPD^**^/FeCl_3_ method [32]
Lemon juice	6.5	0.86	6.25	0.68	7.0 mmoles/ kg fruit (limes) by FRAP^***^ [7] 7.3 mmoles Kg fruit (limes) by FRAP [33]
Fanta orange	0.71	1.03	0.70	0.69	-
Cappy grapefruit	0.064	1.15	0.0615	0.67	-
Frutti fresh Tutti Frutti	4.0	0.732	4.32	0.51	-
Fanta lemon	1.54	0.870	1.45	0.74	2.20 mM lemon soft beverage by ABTS+. method [13]
Prigat orange	2.40	0.790	2.46	0.67	3.02 mM orange soft beverage by ABTS+. method [13]
Prigat peach	1.24	0.795	1.20	0.63	2.51 mM peach soft beverage by ABTS+. method [13]

*The value represents the hydrophilic antioxidant activity (HAA) [32], determined for the aqueous citrus extract; **N,N-dimethyl-*p*-phenylenediamine; ***ferric reducing antioxidant power assay.

For verifying the degree of recovery, known Trolox^®^ amounts (from a more concentrated, 1 M solution, with the exception of Cappy grapefruit, for which a 100 mM Trolox solution was used) were added to the respective juices, which were subsequently analysed by using the experimental conditions described at the working procedure. Due the small volume required from the concentrated Trolox^®^ solution (comprised between 6 and 94 µl) no correction of volume was taken into account for calculating the degree of recovery (see Table 2). As can be seen in Table 1, the results obtained in this study are in good agreement with the previously published data.

**Table 2:** Determination of the degree of recovery of known concentrations of Trolox introduced in the analysed samples, by using the biamperometric method.

*Product*	*Measured TEAC value (mM Trolox) after 1st addition*	*Recovery % after 1^st^ addition*	*Measured TEAC value (mM Trolox) after 2nd addition*	*Recovery % after 2^nd^ addition*
Prigat peach	1.73	101.76	2.16	98.18
Prigat orange	3.50	101.15	4.45	99.77
Fanta lemon	2.0	102.56	2.48	101.22
Fanta orange	0.97	102.10	1.21	100.83
Cappy grapefruit	0.0875	101.15	0.11	98.65
Frutti freshTutti Frutti	6.16	97.47	8.14	97.83
Lemon juice	8.7	99.42	11.2	99.55
Orange juice	13.05	101.80	16.80	101.39

## 3. Conclusions

A sensitive and rapid biamperometric method was designed for monitoring the total antioxidant content in fruit juices. The method has proved its accuracy by the degrees of the recovery obtained for known quantities of Trolox^®^, added to the analysed samples ranging between 97.47 and 102.56%, as well as the results comparable to those of the spectrophotometric method. The precision of the method was proved by the R.S.D. value of 1.29% (n = 10, c = 15 μM Trolox^®^). 

Although the dynamic and linear ranges are more extended in the case of the spectrophotometric method (0.01-0.125 mM linearity for the spectrophotometric method and 5-30 μM for the biamperometric method), the biamperometric method has turned out to be more sensitive, allowing the detection of concentrations as low as 5 µM Trolox^®^. 

The results obtained in this study are in good agreement with the ones previously published in literature, for similar products [7,13,32], as shown by the comparison presented in Table 1. Thus, biamperometry can be successfully used in food industry for assessing the antioxidant content of natural fruit juices and soft drinks, being an accurate, rapid and relatively cheap method. 

## 4. Experimental

### 4.1. Reagents and solutions

2,2-Diphenyl-1-picrylhydrazyl (DPPH·), 2,2-diphenyl-1-picrylhydrazine (DPPH) and Trolox^®^ (Sigma Aldrich). Potassium dihydrogen phosphate and sodium monohydrogen phosphate (Riedel de Haën). Potassium chloride (Sigma Aldrich). Methanol and ethanol (Sigma Aldrich). Trolox^®^ was chosen as water-soluble antioxidant for both methods and a 1mM stock solution was prepared by dissolving the antioxidant in distilled water which was previously boiled and chilled until it reached room temperature. For spectrophotometric experiments, DPPH· was dissolved in methanol as to obtain a stock solution of 0.24 mM concentration. For the working antioxidant solutions, Trolox^®^ concentrations varied between 0.01 and 0.20 mM and were prepared by diluting the stock solution with methanol.

For all chronoamperometric experiments, the DPPH·/DPPH mixture solution was prepared by DPPH· and DPPH dissolution in an ethanolic phosphate buffer solution, so as to reach concentrations of 150 µM DPPH· and 165 µM DPPH, respectively. For the cyclic voltammetry experiments, the DPPH·/DPPH mixture solution of 0.5 mM (each component) was prepared by dissolution of the necessary amount of DPPH·/DPPH in the ethanolic phosphate buffer. The ethanolic phosphate buffer solution was prepared by mixing 0.055 M potassium dihydrogen phosphate with 0.055 M sodium monohydrogen phosphate in an 1/4 ratio in order to reach a pH = 7.40, adding absolute ethanol, up to 40% (v/v) concentration in the final solution, and then solid KCl as supporting electrolyte, up to 0.33 M concentration in the final solution.

### 4.2. Instrumentation

**Figure 8:**
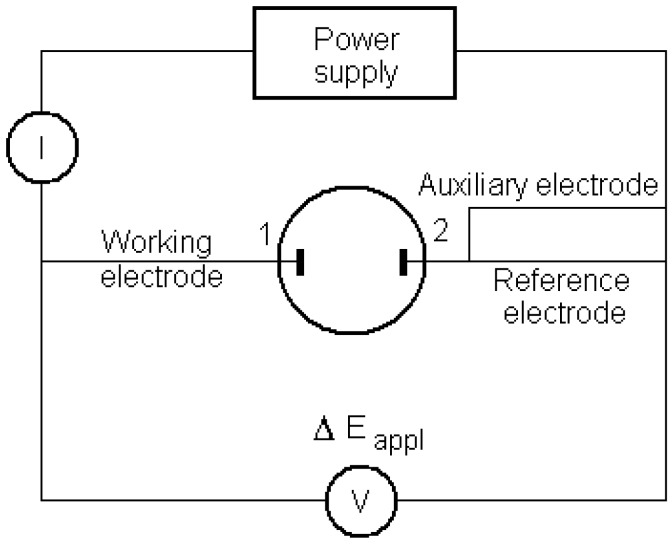
Scheme of the biamperometric system with two identical Pt electrodes (1 and 2) used for measuring the total antioxidant activity; the potentiostat enables measuring of the current intensity (I), at a fixed (applied) voltage (V); electrode 1 is connected to the WORKING ELECTRODE output, while electrode 2 is connected to the REFERENCE and AUXILIARY outputs of the potentiostat.

Spectrophotometer Spekol 11 Carl Zeiss Jena; potentiostat-galvanostat coupled to computer (laboratory-made by Prof. dr. Slawomir Kalinowski, Warmia and Mazury University, Olsztyn, Poland); magnetic stirrer (Metrohm AG); centrifuge (Qualitron, Korea); two identical Pt strip electrodes (Radelkis, 30 mm^2^ surface, mounted in a 25 mL electrochemical cell) for biamperometric determinations. In the case of cyclic voltammetry experiments, these Pt strip electrodes were used as working and auxiliary. A saturated calomel electrode (Radelkis) was introduced as reference. The scheme of the biamperometric system is presented in Figure 8. 

Working procedure: The spectrophotometric method: the value of the absorbance was monitored at 515 nm using a 1 cm spectrophotometric cell. The blank solution (0.12 mM DPPH·) was prepared by diluting the 0.24 mM solution with methanol in 1/1 ratio). The analysed samples were measured versus the blank sample. 10 mL volumetric flasks were used, in which 0.24 mM DPPH· solution (5 mL) and different volumes of 1 mM Trolox^®^ solution (or a volume comprised between 0.12 and 5 mL of the analysed sample) were added, then methanol was added up to 10 mL, so that Trolox5 mL of solution concentrations varied within the range 0.01 mM - 0.20 mM in the final solution. The decrease of DPPH· absorbance was monitored.

In the case of the electrochemical method, 25 mL volumetric flasks were used, to which the indicating mixture solution (16.7 mL) containing 150 µM DPPH· and 165 µM DPPH were first added, so as to have 100 µM and 110 µM concentrations, respectively, in all final solutions. Then, different volumes of 1 mM Trolox^®^ solution were added. The ethanolic phosphate buffer solution was made up to 25 mL volume, so that Trolox^®^ concentrations varied within the range 5 - 30 µM in the final solutions. Before each determination, both Pt electrodes were cleaned electrochemically in 1.25 M H_2_SO_4_ solution by applying four potential pulses of -1.5 V (*versus SCE*) for three seconds and ultrasonically in a water bath for two minutes. All experiments were performed under stirring using a 25 mL electrochemical cell. For the biamperometric experiments we employed a ΔE of 200 mV potential difference between the two electrodes.
